# Hydrocortisone vs. Methylprednisolone in Community-Acquired Pneumonia: A Propensity Score Matching Study

**DOI:** 10.3390/jcm15145725

**Published:** 2026-07-21

**Authors:** Juan Sebastian Hernández Puentes, Alirio Rodrigo Bastidas, Eduardo Andres Tuta Quintero, Catalina Marenco Galvis, Juanita Fetecua Chaparro, Alejandra Mora Vega, Valeria Leyton Franco, María José Juvinao Morales, María José Castro Salas, Juan Sebastián Ariza Zúñiga, Viviana Catalina Andrade, Isabella Criado Quintero, Laura Valentina Medellín Ortiz, Dayanna Beatriz Colpas Echeverri, Luisa Fernánda Arriaga Bustos, Lina María López Nuñez

**Affiliations:** 1 School of Medicine, Universidad de La Sabana, Campus del Puente del Común, Km 7 Autopista Norte de Bogotá, Chía 250001, Cundinamarca, Colombia; 2 Clinical Medicine Applied Research Group, Universidad de La Sabana, Campus del Puente del Común, Km 7 Autopista Norte de Bogotá, Chía 250001, Cundinamarca, Colombia; 3 Department of Internal Medicine, Universidad de La Sabana, Campus del Puente del Común, Km 7 Autopista Norte de Bogotá, Chía 250001, Cundinamarca, Colombia

**Keywords:** pneumonia, hydrocortisone, methylprednisolone

## Abstract

Community-acquired pneumonia is one of the main causes of morbidity and mortality due to infections worldwide. This has led to the study of the use of corticosteroids as adjunctive therapy, which has shown an inclination to provide clinical benefits. However, controversy persists regarding the use of one corticosteroid over another **Objectives:** Evaluate the outcomes associated with the use of hydrocortisone versus methylprednisolone in patients with community-acquired pneumonia. **Methods:** We conducted a multicenter retrospective cohort study that included hospitalized adults diagnosed with CAP at two high-complexity clinics in Colombia between 2010 and 2020. Propensity score matching was used in a 1:1 ratio to balance the baseline characteristics between the hydrocortisone and methylprednisolone groups. The primary outcome was 30-day mortality. Secondary outcomes included septic shock, vasopressor use, admission to the intensive care unit, mechanical ventilation, and other hospital outcomes. Survival analyses were performed using Kaplan–Meier curves and estimates of the average treatment effect, as well as subgroup analyses according to the dose and duration of treatment. **Results:** The study initially included 366 patients, with significant baseline differences between groups. After matching, an adjusted cohort of 168 patients (84 per group) was obtained, with an adequate balance of clinical, paraclinical, and severity-related variables. No statistically significant differences were found in 30-day mortality between patients who received hydrocortisone and those who received methylprednisolone before or after matching. Additionally, no differences were detected in the secondary clinical outcomes. Survival analyses revealed no differences according to corticosteroid type, treatment duration, or administered dose. **Conclusions:** In this retrospective propensity-score-matched cohort study, no differences were observed in 30-day mortality or other clinical outcomes between hydrocortisone and methylprednisolone in patients with community-acquired pneumonia.

## 1. Introduction

Community-acquired pneumonia (CAP) is one of the main causes of morbidity and mortality from infectious diseases worldwide, with an estimated global incidence of 4350/100,000 inhabitants in 2021. Among hospitalized patients, 13–22% have severe community-acquired pneumonia and often require intensive care, with respiratory failure, septic shock, and multiorgan dysfunction driving high mortality rates. From a pathophysiological standpoint, a proportion of severe CAP cases are characterized by a dysregulated inflammatory response, which has driven research into the use of corticosteroids as immunomodulatory adjunctive therapy to antibiotic treatment to impact the fatal outcomes of this disease [1].

Various studies have evaluated the impact of corticosteroids on the treatment of community-acquired pneumonia (CAP), with heterogeneous results depending on the type of steroid used, the clinical severity of patients, and the outcomes evaluated, generating controversy regarding their use. Nevertheless, the most recent evidence tends to show relevant clinical benefits, including a reduction in mortality [2], the requirement for invasive mechanical ventilation, and admission to intensive care units [3] without a significant increase in adverse events [4]. In this context, the CAPE COD clinical trial [5] demonstrated that early administration of hydrocortisone in patients with severe CAP admitted to the ICU was associated with a significant reduction in the 28-day mortality rate.

On the one hand, a meta-analysis by See et al. [6] showed that the use of corticosteroids was associated with an approximately 30% reduction in mortality, with a particularly relevant effect for hydrocortisone, which showed a lower risk of death than other corticosteroids. These findings are consistent with those reported in a systematic review and meta-analysis of randomized controlled trials by Cheema et al. [7]. In contrast, a study published in JAMA in 2015, which included patients with community-acquired pneumonia and an elevated inflammatory response, showed that methylprednisolone was associated with a reduction in treatment failure rates [8].

In contrast, a retrospective analysis of the MIMIC-IV database [9] offers a direct comparison between methylprednisolone and hydrocortisone in critically ill patients with septic shock, a condition that is frequently present in cases of severe CAP. This study found no significant differences in 30-day mortality between the two groups. Similarly, in a study by Sato et al. [10], mortality was similar between the two corticosteroid groups (*p* = 0.054), with no clear differences in secondary outcomes in the general population. Based on the results of various studies, the American Thoracic Society, in its most recent update on the diagnosis and treatment of community-acquired pneumonia, still differs and highlights the need to understand subgroup analyses according to the type of corticosteroid, which may be more effective at reducing mortality [11].

Wang et al. [12] proposed that both the choice of corticosteroid type and the duration of treatment may significantly influence clinical outcomes. Taken together, the available evidence indicates that although corticosteroids play a role in the management of CAP, the selection of a specific molecule could modify outcomes, positioning hydrocortisone as a promising alternative to methylprednisolone. However, there is a lack of studies designed to directly compare these two approaches. Therefore, the objective of the present study was to evaluate the outcomes associated with the use of hydrocortisone versus methylprednisolone in CAP.

## 2. Materials and Methods

A multicenter retrospective cohort study was conducted using propensity score matching (PSM). The study included patients treated at two high-complexity clinics in Colombia between January 2010 and December 2020.

### 2.1. Subjects and Eligibility Criteria

The included subjects were adults older than 18 years with a diagnosis of pneumonia according to ATS criteria [13], acute respiratory symptoms associated with radiological findings compatible with pneumonia and required antibiotic treatment. Patients without a medical record, with incomplete variables in the PSI score [14], with a diagnosis of nosocomial pneumonia during hospitalization, immunosuppressed patients, and those with connective tissue diseases were excluded from the study.

### 2.2. Variables and Data Collection

Information was obtained on demographic characteristics, vital signs, medical history, physical examination findings, and clinical course. Data were collected from electronic medical records, and follow-up and mortality information were obtained from the Colombian National Health Information System (Administradora de los Recursos del Sistema General de Seguridad Social en Salud). The study was conducted according to the type of corticosteroid used: hydrocortisone or methylprednisolone. In addition, a subgroup analysis according to treatment duration (in days) and administered dose was performed to evaluate their possible influence on 30-day mortality using a matched cohort. High doses were defined according to established clinical ranges: ≥300 mg/day for hydrocortisone and ≥80 mg/day for methylprednisolone [15,16]. Clear eligibility criteria were established, and the research team was trained in data collection to reduce the risk of selection and information bias. To minimize data entry errors, the data were reviewed by at least two members of our research team.

### 2.3. Sample Size

Statistical power was calculated a priori to determine the study’s ability to detect clinically relevant differences between groups. Using a total sample size of 168 patients, equally distributed into two independent groups (n = 84), and setting a significance level alpha of 0.05, the study achieved a statistical power of 95.8%. This calculation was based on an expected difference of 30 percentage points in the primary outcome (proportions of 50% vs. 20%). The analysis was performed using a Z-test to compare two independent proportions [17].

### 2.4. Statistical Analysis

Data were extracted directly from electronic medical records, which were fully reviewed and transferred to the electronic data capture software, Research Electronic Data Capture (REDCap, Version 16 LTS). Subsequently, the data were exported to Excel and analyzed using Python. Categorical variables were summarized as frequencies and percentages, quantitative variables as mean and standard deviation if normally distributed, and median and interquartile range if not normally distributed. Categorical variables were compared using the chi-square test, and continuous variables were compared using Student’s *t*-test for independent samples or the Mann–Whitney U test, depending on their distribution.

Propensity score matching (PSM) analysis was performed to reduce selection bias due to differences in baseline characteristics between patients who did and did not receive corticosteroids. The balance between the treated and untreated groups was evaluated using the standardized mean difference. A propensity score was estimated using a logistic regression model that matched patients in a 1:1 ratio.

Matching was performed using the nearest-neighbor matching (NNM) method. Covariate balance before and after matching was assessed using standardized mean differences and Rubin’s B statistic to ensure comparability between the treatment and control groups. The variables included in the propensity score model were selected based on biological plausibility and statistical significance and comprised pleural effusion, arterial partial pressure of carbon dioxide (PaCO_2_), mean arterial pressure at admission, Pneumonia Severity Index (PSI) score, sex, age, heart rate at admission, systolic blood pressure at admission, smoking status, chronic heart failure, arterial oxygen-to-inspired oxygen ratio (PaO_2_/FiO_2_), serum creatinine, alveolar infiltrates, and the use of non-invasive ventilation. After matching, the average treatment effect (ATE) and average treatment effect on the treated (ATET) were estimated, along with their corresponding 95% confidence intervals (95% CIs). Kaplan–Meier survival curves were generated before and after matching to evaluate differences in survival between groups. Statistical significance was defined as a two-sided *p*-value < 0.05.

## 3. Results

### 3.1. General Characteristics

**Figure A1:**
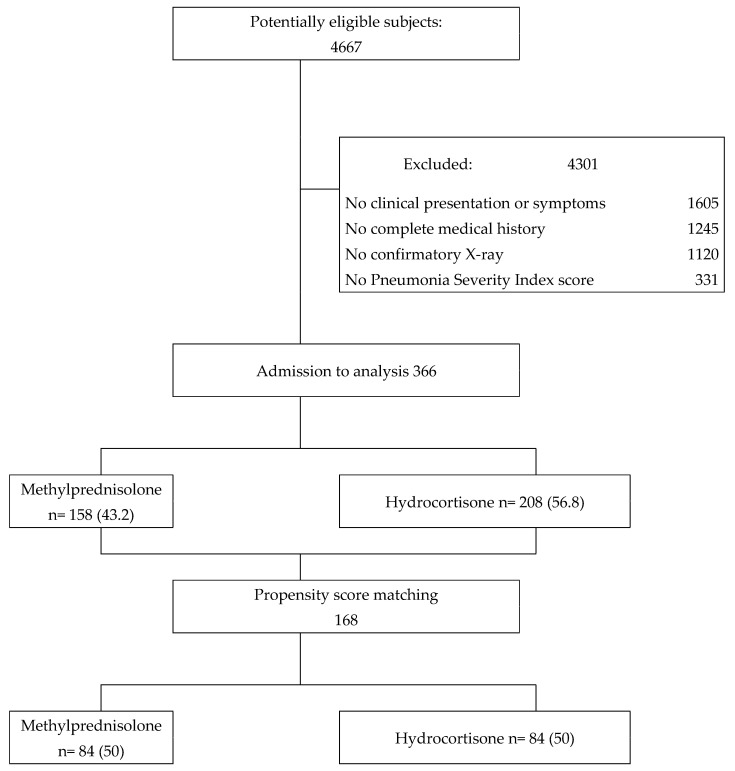
Flowchart of subject admission to the study.

In the original cohort of 366 patients (Figure A1), the mean age was 68.2 years, and males predominated (55.2%). When comparing the treatment groups, patients who received hydrocortisone were younger than those treated with methylprednisolone (66.3 vs. 70.6 years; *p* < 0.001). Additionally, they had lower oxygen saturation on admission (86.9% vs. 88.5%; *p* < 0.001) and required a lower initial FiO_2_ (28.6% vs. 31.7%; *p* < 0.001).

After the propensity score matching process, these differences between groups were completely mitigated: in the adjusted cohort of 168 patients (84 per group), all variables—general, clinical, and medical history—showed *p*-values greater than 0.05 and standardized differences < 0.1. No significant differences were found either before or after matching (Table 1).

### 3.2. Paraclinical Variables

In the original cohort of paraclinical variables, significant differences were also observed before matching. First, hemoglobin levels in patients who received hydrocortisone were lower than those in patients who received methylprednisolone (13.5 vs. 13.9 g/dL; *p* = 0.038). Regarding renal function, creatinine levels were higher in the hydrocortisone group (1.4 vs. 1.1 mg/dL; *p* < 0.001). With respect to arterial blood gases, prior to matching, most evaluated values showed significant differences; the pH was lower in patients treated with hydrocortisone (*p* = 0.014). Additionally, the PaO_2_/FiO_2_ ratio was lower in the group that received methylprednisolone (203 vs. 217.9; *p* < 0.001).

Regarding radiographic findings, there were significant differences in the evaluation of certain patterns of involvement, with patients receiving hydrocortisone more frequently showing alveolar infiltrates (58.2% in methylprednisolone vs. 72.6% in hydrocortisone; *p* = 0.004), atelectasis (*p* < 0.001), consolidation (51.3% vs. 70.7%; *p* < 0.001), and multilobar involvement (27.8% vs. 40.4%; *p* = 0.013) than those receiving methylprednisolone. After matching, variations in paraclinical variables disappeared, and no statistically significant differences were observed between the groups (Table 2).

### 3.3. Clinical Outcomes

In terms of clinical outcomes, a difference was identified in the original cohort, showing that septic shock was more frequent in patients treated with hydrocortisone (23.6% vs. 15.2%; *p* = 0.047). However, other outcomes, including vasopressor support, ICU admission, invasive or noninvasive mechanical ventilation, hospitalization, remission, and in-hospital mortality, did not differ significantly between the treatment groups. Specifically, mortality rates were 11.5% and 10.1% in the hydrocortisone and methylprednisolone groups, respectively (*p* = 0.668). After propensity score matching, the observed differences were attenuated, and none of the evaluated clinical outcomes were statistically significant. Mortality in the matched cohort was 19.6% among patients receiving hydrocortisone compared with 10.7% in those receiving methylprednisolone; however, this difference did not reach statistical significance (*p* = 0.162) (Table 3).

#### Survival Description

**Figure 1:**
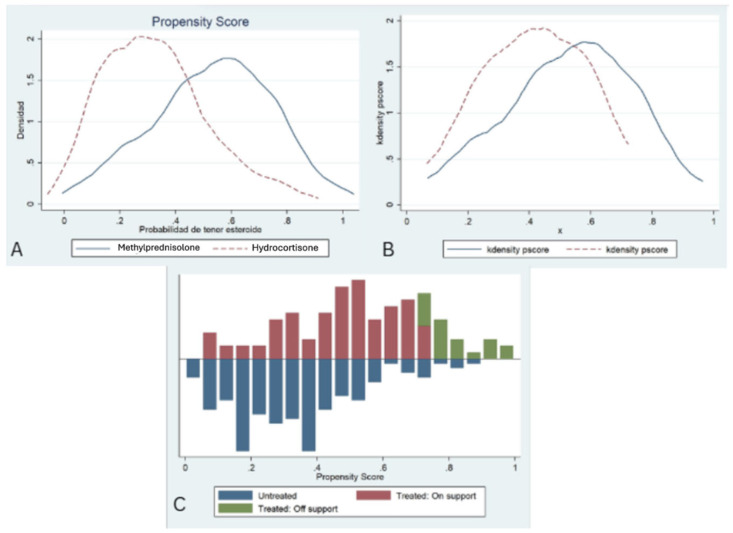
Distribution of the propensity score before and after matching between patients who received methylprednisolone and those who received hydrocortisone. Distribution before (**A**) and after (**B**) propensity score matching between patients who received methylprednisolone and those who received hydrocortisone. (**C**) Region of common support between patients who received methylprednisolone and those who received hydrocortisone.

The propensity score distributions showed an imbalance before matching, which was notably reduced after matching, with a greater overlap between groups and an adequate region of common support. Additionally, the histogram confirmed that most observations remained within the support, with only a few cases excluded (Figure 1).

**Figure 2:**
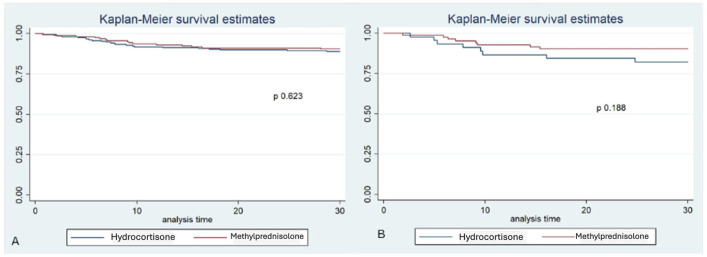
Kaplan–Meier curves before and after matching between patients who received methylprednisolone and those who received hydrocortisone. Kaplan–Meier survival curves comparing mortality between patients who received methylprednisolone and those who received hydrocortisone, before (**A**) and after (**B**) propensity score matching.

**Figure 3:**
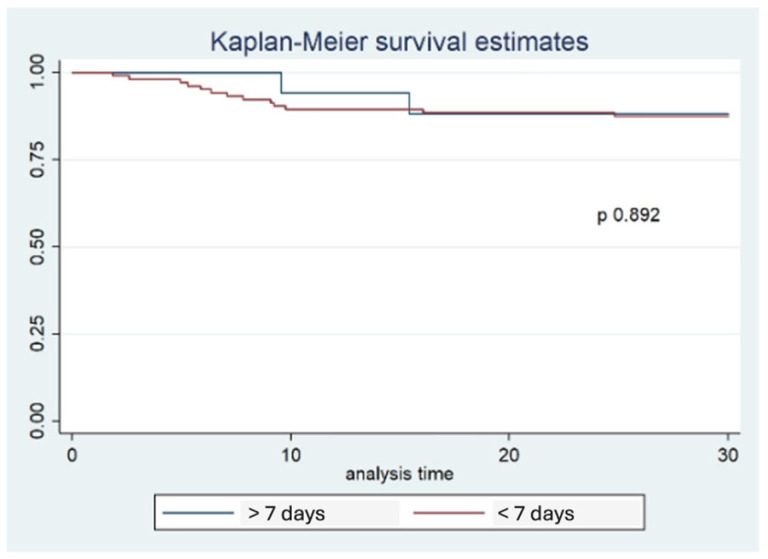
Kaplan–Meier survival curve at 30 days according to duration of corticosteroid treatment.

**Figure 4:**
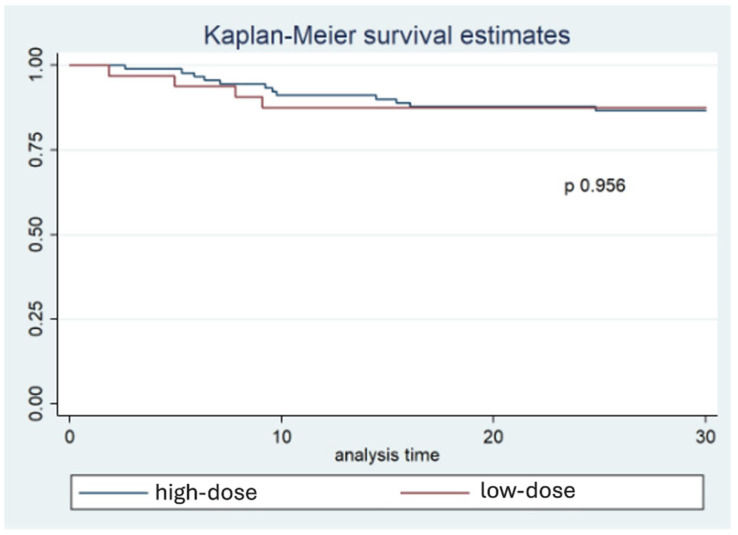
Kaplan–Meier survival curve at 30 days according to corticosteroid treatment dose.

A 30-day survival analysis was performed to evaluate the differences between the use of hydrocortisone and methylprednisolone, and no significant differences were found either before (*p* = 0.623) or after matching (*p* = 0.18) (Figure 2). Thirty-day survival was also evaluated according to the duration of corticosteroid treatment, without evidence of differences between use for less than 7 days and >7 days (*p* = 0.892) (Figure 3). Additionally, survival was analyzed according to the administered dose, with no differences between high- and low-dose corticosteroid treatments (*p* = 0.956) (Figure 4). No statistical significance was observed in any of the estimation methods, including ATE, ATT, and ATET (Table 4).

## 4. Discussion

This study evaluated 30-day mortality between the use of hydrocortisone versus methylprednisolone in patients with community-acquired pneumonia, using the propensity score matching method, where in the original cohort, relevant baseline differences were found between the groups, including age, oxygenation parameters, renal function, radiographic findings, and a higher frequency of septic shock in the hydrocortisone group. These inequalities were adequately balanced after the matching process, allowing for a more homogeneous comparison between the two therapeutic strategies. In this context, survival analyses and effect estimates (ATE, ATT, and ATET) consistently demonstrated the absence of a mortality benefit associated with either corticosteroid, regardless of dose or duration of treatment.

These findings are consistent with those of previous studies that compared the use of different systemic corticosteroids in the management of community-acquired pneumonia. In a retrospective analysis based on the MIMIC-IV database conducted by Xu et al. [9], no significant differences were observed in clinical outcomes between patients treated with methylprednisolone and those who received hydrocortisone. This similarity in results could be explained, in part, by the clinical equivalence of glucocorticoids when administered at equipotent doses, given that they share mechanisms of action mediated by the glucocorticoid receptor and comparable final anti-inflammatory effects.

Although there are pharmacokinetic and pharmacodynamic differences between the two drugs, including plasma half-life, receptor affinity, and duration of biological effect [18,19], these variations do not necessarily translate into clinically relevant differences in outcomes, such as mortality, when the therapeutic objective is to achieve systemic modulation of the inflammatory response. In this context, it is plausible that the observed clinical impact depends more on overall inflammation control and timing of administration than on the specific corticosteroid used, as previously suggested in pharmacodynamic models and comparative clinical studies [20].

In the original analysis, hydrocortisone was used more frequently in patients with septic shock, which could be explained by its historical role as the corticosteroid of choice in this scenario, as it has been the most widely studied in critically ill patient populations [21]. Accordingly, the most recent update of the Surviving Sepsis Campaign recommends the use of hydrocortisone for patients with refractory septic shock [22], given that most clinical trials conducted in this context have employed this drug alone.

Additionally, this preference could be explained by the results of some studies that have suggested a possible advantage of hydrocortisone in terms of mortality reduction compared with other corticosteroids [12,23]; however, these findings are inconsistent with the results of the present study. Both meta-analyses highlight the need for additional studies to provide more precise guidance for clinical decision-making.

Although hydrocortisone was used more frequently in patients with septic shock, this finding likely reflects its usual use in a specific critically ill patient scenario rather than selection based on pneumonia severity. Indeed, when disease severity was analyzed using the Pneumonia Severity Index (PSI), no clear preference for one or the other corticosteroid was observed before or after propensity score matching. The absence of differences according to the PSI is relevant, considering that this scale has been validated not only as a prognostic tool but also as a support for clinical decision-making [14]. In this context, the results suggest that outside the septic shock scenario, the choice between hydrocortisone and methylprednisolone in community-acquired pneumonia likely depends more on the treating physician’s judgment than on an objective stratification of severity.

In the outcomes evaluated subsequently, no statistically significant differences were identified between the groups, including vasopressor use, admission to the intensive care unit, need for invasive or noninvasive mechanical ventilation, length of hospitalization, or clinical remission. These findings suggest that the choice of a specific corticosteroid may not have a clinically relevant impact on the management of community-acquired pneumonia and that the decision to use one or the other may be primarily determined by the clinician’s preference. This result is consistent with that reported by Sato et al. [10], who also found no significant differences when comparing both corticosteroids.

Although the findings of this study are consistent with the current literature, several limitations must be acknowledged. First, its retrospective observational design based on electronic medical records carries an inherent risk of selection bias and residual confounding, despite the use of propensity score matching (PSM). In addition, corticosteroid selection may have been influenced by disease severity and institutional prescribing practices, introducing potential indication bias, particularly in patients with septic shock. Although the Pneumonia Severity Index was included as a global measure of disease severity, the sample size and data structure did not permit subgroup analyses according to severity, which may have identified populations deriving differential benefits from a specific corticosteroid regimen. Furthermore, standardized data on inflammatory biomarkers, microbiological etiology, and antibiotic appropriateness were not available. Information on corticosteroid-related adverse events, including hyperglycemia, secondary infections, gastrointestinal bleeding, delirium, and other treatment-related complications, was not systematically collected in the clinical records and, therefore, could not be analyzed. Similarly, data on hospital readmissions were not available. Finally, follow-up was limited to 30 days, precluding assessment of longer-term outcomes and the safety profile associated with corticosteroid therapy.

## 5. Conclusions

In conclusion, the present study did not demonstrate the superiority of hydrocortisone over methylprednisolone, suggesting that both corticosteroids could be used interchangeably in the treatment of community-acquired pneumonia at the treating physician’s discretion. Nevertheless, these findings should be interpreted with consideration of the limitations inherent to the study design, as well as the need for randomized clinical trials to confirm these observations and provide guidance for clinical decision-making.

## Figures and Tables

**Table 1:** General characteristics of the population.

	Original Cohort				Matched Cohort				Standardized Difference	
	Total Population n = 366	Methylprednisolone n = 158	Hydrocortisone n = 208	*p* Value	Total Population n = 168	Methylprednisolone n = 84	Hydrocortisone n = 84	*p* Value	Unmatch n = 366	Match n = 168
Age, years	68.2 (20.06)	70.6 (19.91)	66.3 (20.02)	<0.001	68.5 (19.17)	68.5 (20.03)	68.6 (17.73)	1.000	0.005	0.122
Male sex	202 (55.2)	89 (56.3)	113 (54.3)	0.703	87 (51.7)	43 (51.2)	44 (52.2)	0.915	0.028	0.014
Clinical symptoms n (%)										
Cyanosis	45 (12.3)	20 (12.7)	25 (12)	0.854	26 (15.7)	10 (11.9)	16 (19.6)	0.237	0.009	0.106
Chest retractions	138 (37.7)	61 (38.6)	77 (37)	0.756	66 (39.1)	31 (36.9)	35 (41.3)	0.622	0.023	0.062
Headache	19 (5.2)	9 (5.7)	10 (4.8)	0.704	8 (4.7)	6 (7.1)	2 (2.2)	0.230	0.013	0.072
Altered level of consciousness	56 (15.3)	27 (17.1)	29 (13.9)	0.408	24 (14.4)	15 (17.9)	9 (10.9)	0.291	0.045	0.101
Crackels	226 (61.7)	97 (61.4)	129 (62)	0.903	110 (65.2)	53 (63.1)	57 (67.4)	0.624	0.009	0.061
Wheezing	156 (42.6)	60 (38)	96 (46.2)	0.117	70 (41.9)	32 (38.1)	38 (45.7)	0.402	0.115	0.106
Vital signs x (SD *)										
Mean arterial pressure	88.3 (16.54)	89.5 (15.21)	87.3 (17.47)	0.068	88.6 (17.54)	89.2 (14.54)	87.5 (22.13)	0.801	0.137	0.405
Respiratory rate	23.2 (6.05)	23.6 (5.99)	22.9 (6.09)	0.167	23.3 (6.32)	23.2 (6.11)	23.5 (6.76)	1.000	0.016	0.102
Temperature	36.7 (0.86)	36.7 (0.87)	36.8 (0.85)	1.000	36.8 (0.83)	36.8 (0.89)	36.8 (0.73)	1.000	0.020	0.185
Oxygen saturation	87.5 (9.23)	88.5 (7.01)	86.9 (10.24)	<0.001	88.4 (7.62)	88.1 (7.22)	88.9 (8.27)	0.537	0.368	0.135
FiO_2_ at admission	29.9 (16.01)	31.7 (16.88)	28.6 (15.21)	<0.001	30.5 (16.41)	30.7 (16.22)	30 (16.92)	1.000	0.104	0.042
Medical history										
Arterial Hypertension	205 (56)	88 (55.7)	117 (56.3)	0.916	54 (59.1)	27 (59.5)	27 (58.7)	0.927	0.008	0.012
Smoking	96 (26.2)	28 (17.7)	68 (32.7)	0.001	25 (27.1)	11 (23.8)	14 (30.4)	0.411	0.204	0.092
Chronic heart failure	58 (15.8)	17 (10.8)	41 (19.7)	0.020	9 (9.7)	5 (10.7)	4 (8.7)	0.714	0.123	0.029
Acute myocardial infarction	19 (5.2)	6 (3.8)	13 (6.3)	0.295	6 (6.2)	3 (6)	3 (6.5)	0.897	0.034	0.008
COPD +	166 (45.4)	77 (48.7)	89 (42.8)	0.258	37 (40.4)	19 (41.7)	18 (39.1)	0.778	0.084	0.036
Diabetes Mellitus	54 (14.8)	29 (18.4)	25 (12)	0.091	16 (17.7)	9 (20.2)	7 (15.2)	0.480	0.092	0.072
Malignancy	14 (3.8)	3 (1.9)	11 (5.3)	0.094	3 (3.4)	1 (2.4)	2 (4.3)	0.535	0.047	0.028
Dementia	28 (7.7)	8 (5.1)	20 (9.6)	0.105	8 (8.9)	2 (4.8)	6 (13)	0.090	0.063	0.113
Asthma	15 (4.1)	7 (4.4)	8 (3.8)	0.780	4 (4.6)	2 (4.8)	2 (4.3)	0.914	0.008	0.006
PSI °	96.8 (38.57)	98.4 (35.02)	95.6 (41.1)	0.991	97.3 (37.97)	98.2 (36.1)	95.7 (41.54)	0.999	0.159	0.140

* SD: Standard deviation; + COPD: chronic obstructive pulmonary disease; ° PSI: Pulmonary Score Index.

**Table 2:** Laboratory findings.

	Original Cohort				Matched Cohort				Standardized Difference	
	Total Population n = 366	Methylprednisolone n = 158	Hydrocortisone n = 208	*p* Value	Total Population n = 168	Methylprednisolone n = 84	Hydrocortisone n = 84	*p* Value	Unmatch n = 366	Match n = 168
Hb *	13.7 (2.66)	13.9 (2.59)	13.5 (2.71)	0.038	14 (2.69)	14.1 (2.41)	13.8 (2.82)	0.077	0.0450	0.1659
Hematocrit	41.2 (7.75)	41.4 (7.49)	40.9 (7.96)	0.999	42 (6.56)	41.9 (6.18)	42.1 (7.26)	0.999	0.0609	0.1605
Platelets	252 (104)	249 (96)	254(110)	1.000	246 (96)	245(91)	247 (105)	1.000	0.1300	0.1444
Creatinine	1.3 (1.67)	1.1 (0.52)	1.4 (2.15)	<0.001	1.1 (0.66)	1.1 (0.57)	1.1 (0.81)	1.000	1.0427	0.3325
BUN **	26 (19.97)	25.9 (18.49)	26 (21.07)	1.000	23.5 (14.45)	23.9 (13.16)	22.6 (16.68)	0.860	0.1303	0.2340
Arterial blood gases										
Ph	7.39 (0.09)	7.4 (0.11)	7.39 (0.08)	0.014	7.4 (0.11)	7.4 (0.13)	7.4 (0.08)	1.000	0.2697	0.5232
PO_2_	63.4 (21.75)	62.2 (18.58)	64.3 (23.83)	0.626	62.6 (18.04)	62.7 (19.43)	62.6 (15.37)	1.000	0.2453	0.2316
PCO_2_	36.5 (11.24)	37.8 (12.01)	35.6 (10.59)	<0.001	36.9 (11.04)	36.7 (11.52)	37.2 (10.22)	1.000	0.1254	0.1196
HCO_3_	21.9 (3.39)	23.7 (3.08)	20.9 (3.19)	<0.001	23.4 (2.76)	23.4 (3.34)	23.4 (1.78)	1.000	0.0341	0.5850
BE ***	−2.4 (5.82)	−1.3 (5.12)	−3.2 (6.18)	<0.001	−2.1 (4.91)	−2.1 (4.82)	−2.1 (5.16)	1.000	0.1870	0.0698
PaO_2_/FiO_2_	211.6 (75.16)	203 (79.69)	217.9 (71.24)	<0.001	214.4 (76.34)	211.4 (79.67)	219.9 (70.37)	0.289	0.1117	0.1236
chest X-ray										
Infiltrado intersticial	186 (50.8)	73 (46.2)	113 (54.3)	0.124	85 (50.9)	38 (45.2)	47 (56.5)	0.219	0.115	0.160
Infiltrados alveolares	243 (66.4)	92 (58.2)	151 (72.6)	0.004	117 (69.9)	59 (70.2)	58 (69.6)	0.936	0.208	0.010
Atelectasias	31 (8.5)	9 (5.7)	22 (10.6)	<0.001	17 (10.1)	6 (7.1)	11 (13)	0.266	0.068	0.082
Consolidación	228 (62.3)	81 (51.3)	147 (70.7)	<0.001	101 (59.9)	44 (52.4)	57 (67.4)	0.098	0.280	0.216
Multilobar	128 (35)	44 (27.8)	84 (40.4)	0.013	74 (43.9)	30 (35.7)	44 (52.2)	0.069	0.174	0.230

* HB: Hemoglobin: ** BUN: Blood Urea Nitrogen; *** BE: Base excess.

**Table 3:** Clinical outcomes.

	Original Cohort				Matched Cohort				Standardized Absolute Difference	
	Total Population n = 366	Methylprednisolone n = 158	Hydrocortisone n = 208	*p* Value	Total Population n = 168	Methylprednisolone n = 84	Hydrocortisone n = 84	*p* Value	Unmatch n = 366	Match n = 168
Septic shock	73 (19.9)	24 (15.2)	49 (23.6)	0.047	43 (25.4)	19 (22.6)	24 (28.3)	0.475	0.115	0.079
Vasopressor support	74 (20.2)	27 (17.1)	47 (22.6)	0.194	42 (24.9)	20 (23.8)	22 (26.1)	0.773	0.077	0.032
ICU *	103 (28.1)	45 (28.5)	58 (27.9)	0.900	63 (37.2)	37 (44)	26 (30.4)	0.129	0.008	0.196
IMV +	83 (22.7)	33 (20.9)	50 (24)	0.476	53 (31.3)	27 (32.1)	26 (30.4)	0.841	0.044	0.024
NIMV °	42 (11.5)	24 (15.2)	18 (8.7)	0.052	20 (12)	11 (13.1)	9 (10.9)	0.712	0.095	0.032
Hospitalization	341 (93.2)	151 (95.6)	190 (91.3)	0.113	154 (91.6)	79 (94)	75 (89.1)	0.314	0.059	0.068
Mortality	40 (10.9)	16 (10.1)	24 (11.5)	0.668	25 (15)	9 (10.7)	16 (19.6)	0.162	0.020	0.121

* ICU: Intensive Care Unit; + IMV: Invasive mechanical ventilation; ° NIMV: Non-invasive mechanical ventilation.

**Table 4:** Propensity Score Matching—Treatment effect estimation.

Method/Estimator	Type	Coefficient	95% CI		*p* Value
psmatch2 + Bootstrap (50 reps)	ATT	−0.0833	−0.2766	0.1100	0.398
teffects psmatch	ATE	−0.0476	−0.1399	0.0447	0.312
teffects psmatch	ATET	−0.0673	−0.1914	0.0568	0.288
teffects nnmatch (Mahalanobis)	ATE	−0.0635	−0.1459	0.0189	0.131
teffects nnmatch (Mahalanobis)	ATET	−0.0288	−0.1433	0.0857	0.621
Regression Adjustment (RA)	ATE	0.0081	−0.0839	0.1002	0.863
Regression Adjustment (RA)	ATET	−0.0454	−0.1493	0.0586	0.392
IPW	ATE	−0.0254	−0.1196	0.0688	0.597
IPW	ATET	−0.0434	−0.1475	0.0607	0.414

ATT: Average Treatment Effect on the Treated; ATE: Average Treatment Effect; ATET: Average Treatment Effect on the Treated; IPW: Inverse Probability Weighting; RA: Regression Adjustment; CI (IC): Confidence Interval; psmatch2: Stata 17 command for propensity score matching; teffects: Stata command for treatment effects estimation; Mahalanobis: Mahalanobis distance.

## Data Availability

We confirm that the dataset underlying this study is held and managed by the Clinical Medicine Applied Research Group (Code: COL0084256), affiliated with the Universidad de La Sabana. The extraction, use, and management of these data were explicitly authorized by the institutional Research Ethics Committee, which approved the study protocol and designated this research group as the responsible entity for data handling and custody. The group is committed to ensuring secure and confidential data management and to evaluating any external data access requests in accordance with institutional, legal, and ethical standards. More information about the group can be found on the official MinCiencias GRUPLAC platform (https://scienti.minciencias.gov.co/gruplac/jsp/visualiza/visualizagr.jsp?nro=00000000007713 (accessed on 10 September 2025)). At present, the group’s director is Alirio Rodrigo Bastidas Goyes, who may be contacted at alirio.bastidas@unisabana.edu.co for data access inquiries. In the event of any changes to leadership or contact information, the GRUPLAC page will be updated to ensure continued accessibility.

## Abbreviations

The following abbreviations are used in this manuscript:

CAP	Community-Acquired Pneumonia
PSM	Propensity Score Matching
PSI	Pneumonia Severity Index
ATS	American Thoracic Society
ICU	Intensive Care Unit
ATE	Average Treatment Effect
ATT	Average Treatment Effect on the Treated
NNM	Nearest-Neighbor Matching
CI	Confidence Interval
REDCap	Research Electronic Data Capture
FiO_2_	Fraction of Inspired Oxygen
MIMIC-IV	Medical Information Mart for Intensive Care IV
NEJM	New England Journal of Medicine
JAMA	Journal of the American Medical Association
